# The importance of wearing a seatbelt correctly – A case report of blunt trauma causing complete shearing transection of the gastroduodenal junction

**DOI:** 10.1016/j.ijscr.2020.05.008

**Published:** 2020-05-19

**Authors:** Matheesha Herath, Peter Bautz, Dominic Parker, Christopher Dobbins

**Affiliations:** The Royal Adelaide Hospital, Port Road, Adelaide, South Australia, 5000, Australia

**Keywords:** Trauma, Road traffic accident, Seatbelt, Emergency surgery, Upper gastrointestinal injury

## Abstract

**Introduction:**

Global mortality as a result of road traffic accidents (RTA) has reduced significantly since mandatory implementation of seatbelts. Whilst seatbelt related injury, or “seatbelt syndrome,” is a recognised phenomenon, unrestrained passengers have considerably worse survival outcomes. Improper positioning of seatbelts, as is discussed in the following case, can cause extensive injury.

**Presentation of case:**

Our patient is a 35-year-old female who was a restrained front seat passenger in a car vs. tree collision at 80 km/h. Her seat belt was worn with the shoulder strap under her left axilla. She sustained multiple injuries including complete transection of the gastroduodenal junction. In addition to this she had splenic, liver, transverse colonic, left lower rib and humeral injury. She underwent damage control laparotomy with splenectomy; re-look with gastrojejunostomy and transverse colonic resection with defunctioning ileostomy. She made a good recovery and was discharged after a 4 week admission.

**Discussion:**

Improperly worn seatbelts redistribute decelerative forces to sensitive regions. A multidisciplinary approach is required to effectively manage complex multi-system trauma. In trauma the simplest reconstructive measures can be the most effective and minimise risk of further complications for the patient.

**Conclusion:**

Improperly worn seatbelts pose a significant risk to patients. A traumatic complete gastroduodenal transection can be effectively reconstructed with gastrojejunostomy anastomosis.

## Highlights

• Seat belt related injuries can cause significant morbidity and mortality in road traffic accidents.
• We present a case where an improperly worn seatbelt caused traumatic shearing transection of the gastroduodenal junction.
• A systematic multidisciplinary assessment approach enabled all injuries to be identified early.
• The patient underwent damage control exploratory laparotomy then delayed reconstruction of the alimentary tract.

## 1 Introduction

Road traffic accidents (RTA) are responsible for 1.4 million deaths annually [1]. This equates to 2.5% of global mortality [1]. Trauma is the leading cause of death among young people and despite developments in automotive safety the road traffic mortality rate is increasing [1,2]. Trauma accounts for 30% of intensive care admissions in America and is currently the 3^rd^ leading cause of permanent disability [2,3]. By 2030 trauma is expected to be the leading cause of permanent disability worldwide [4]. Australian data is consistent with global trends demonstrating increasing morbidity and mortality due to RTA [5].

Rudimentary seatbelts were first developed in the late 1800’s for the purpose of air travel [6]. These evolved into automotive use over decades and the 3 point harnesses found in every modern car were first installed in a Volvo in 1959 [7,8]. Australian legislation was implemented in the early 1970’s and standardised seat belt fitting and wearing became mandatory [9]. This coupled with public education campaigns resulted in a significant reduction in RTA related mortality and morbidity at this time [9].

3 point harnesses are designed to distribute the force of deceleration to the clavicle, sternum and pelvis. Cervico-thoracic spinal injury, sternal fracture, and pelvic fracture were thought to be initially associated with these restraints. Evidence demonstrated that unrestrained passengers in similar RTA’s had significantly worse injury and outcomes [8,[10], [11], [12]]. Presence of soft tissue injury as a result of a seat belt, known as “seat belt sign,” is a predictor of further underlying internal injury and is a diagnostic aid to clinicians managing trauma [10,[13], [14], [15], [16], [17]]. An improperly worn seatbelt, as will be described in the following case, can cause significant injury.

## 2 Presentation of case

Our patient is a 35-year-old female. She was the restrained front seat passenger in a car vs. tree RTA at 80 km/h. Her seatbelt was worn with the shoulder strap sitting under her left axilla. The car had no airbags and was right hand drive. The accident occurred in urban South Australia. The 2 other occupants sustained minor injuries. She had a past surgical history of only bilateral breast implants, took no regular medications and had no pre-existing medical conditions. She was heavily intoxicated at the time of the RTA. She denied any previous drug use. She has no significant family or psychiatric history.

**Image 1:**
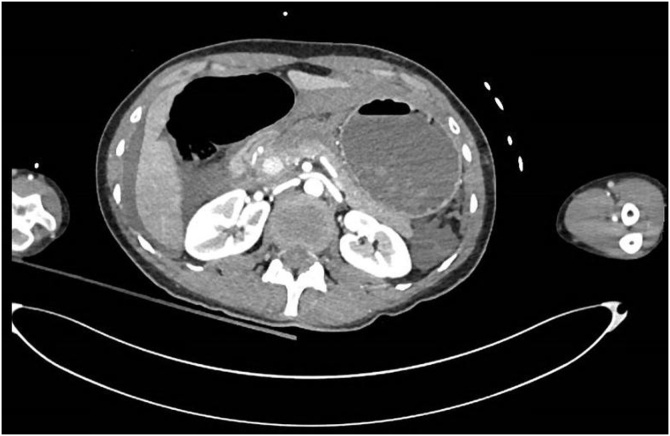
Axial CT demonstrating a distended stomach and free intra-abdominal fluid.

**Image 2:**
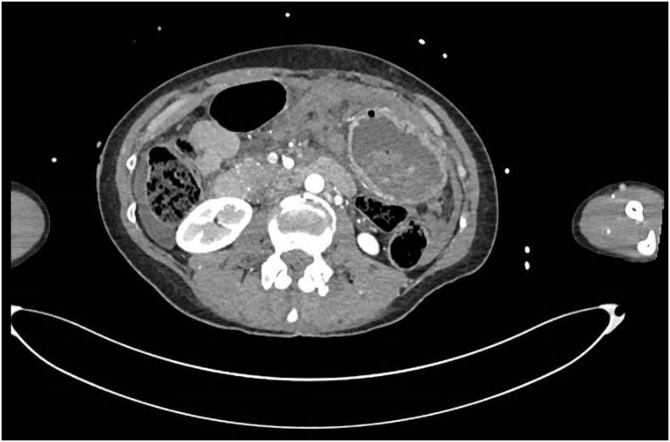
Axial CT demonstrating free gastric content in the peritoneal cavity.

**Image 3:**
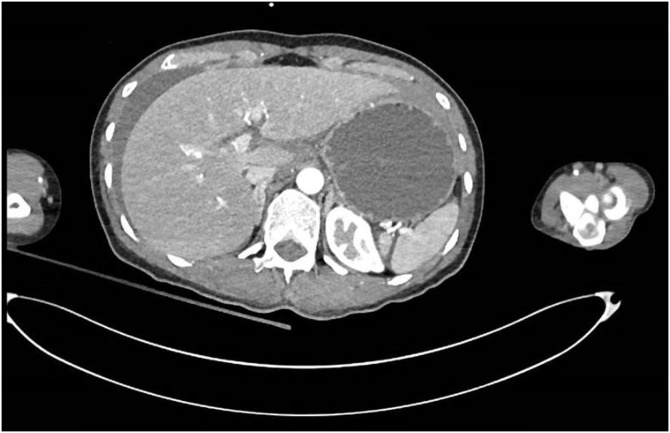
Axial CT showing free fluid around the liver.

**Image 4:**
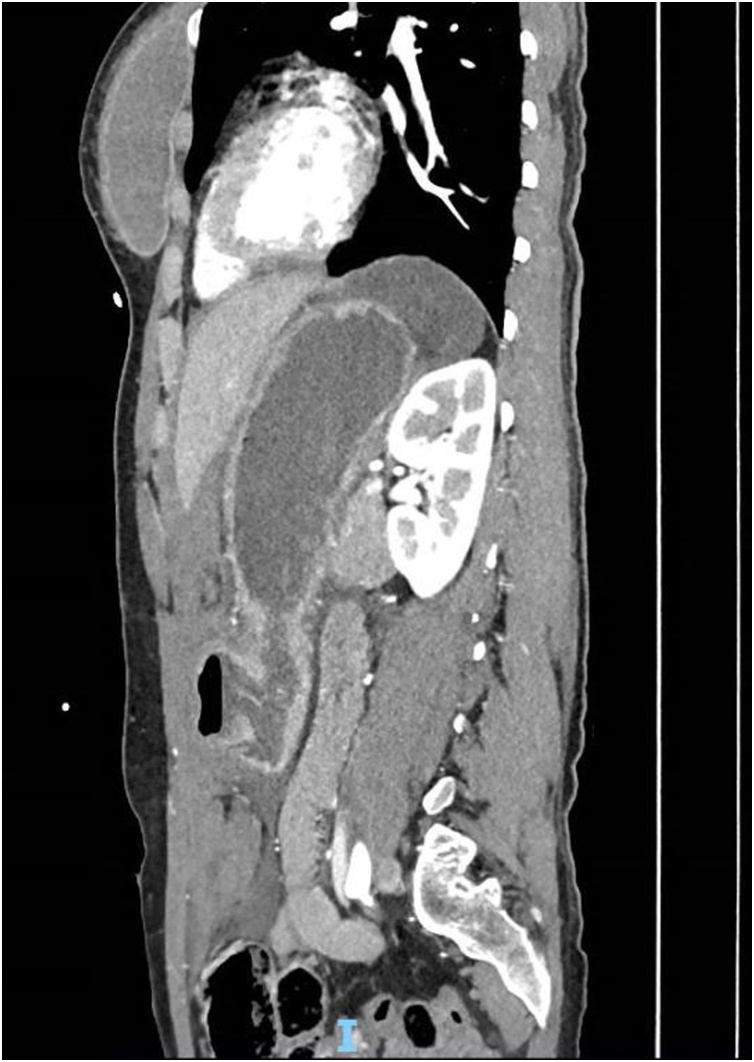
Sagittal CT demonstrating pyloric and duodenal discontinuity with small volume free air just beyond the pylorus.

**Image 5:**
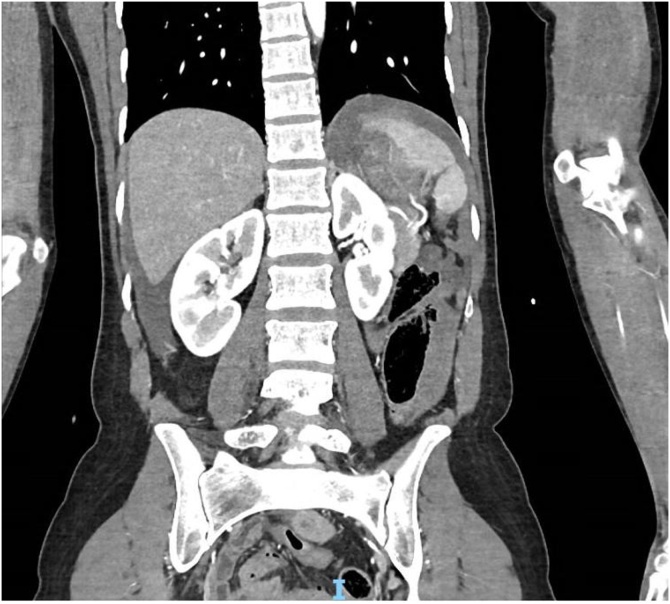
Coronal CT demonstrating splenic laceration with surrounding free fluid.

She was retrieved from the scene to a tertiary trauma centre via ambulance. Primary survey showed a patent airway, equal air entry, tachycardia of 110 bpm and blood pressure of 130/60. She had multiple superficial abrasions, a generally tender abdomen, an unremarkable chest X-ray, and a negative Focused Assessment of Sonography in Trauma (FAST) scan. She was taken immediately for Computer Tomography (CT) imaging from the trauma bay. Imaging showed a moderate volume of free intra-abdominal fluid (Image 1), a distended stomach with transection of the gastroduodenal junction (Image 2, Image 3, Image 4), and splenic injury (Image 5). Upon transfer from CT machine to barouche the patient became haemodynamically unstable with a blood pressure of 80/40 and tachycardia at 130. She was transfused with red blood cells and taken immediately to the operating theatre for exploratory laparotomy.

The patient underwent Rapid Sequence Induction (RSI) and midline laparotomy was performed. The on-call Trauma Surgical Consultant performed the procedure with the on-call Surgical Registrar assisting. She required 10 min of supracoeliac aortic compression until haemodynamic stability was secured. Subcapsular splenic injury with uncontrollable bleeding was identified and managed with splenectomy. Bleeding from a posterior paracaval liver laceration was controlled with packing. Once rapid bleeding was controlled systematic examination of the abdominal viscera revealed a complete shearing transection of the gastroduodenal junction. The pyloric sphincter was seen periodically relaxing and spurting alcohol smelling gastric contents into the abdomen. Pylorus and duodenum were stapled off and an orogastric tube was placed. Further examination showed a 13 cm seromuscular injury of the transverse colon with associated contusion. This was repaired primarily. Systematic examination incorporating all viscera including retroperitoneal structures did not demonstrate further injury. Extensive washout was performed, and the patient’s abdomen was packed with a negative pressure dressing. She was transferred to the Intensive Care Unit (ICU) still intubated and sedated with a view to re-look laparotomy in 48 h.

Secondary survey and imaging identified an L3 chance fracture, multiple lower left sided rib fractures and a humeral fracture. She remained stable in the ICU and was managed with intravenous broad-spectrum antibiotics and received a total of 8 units of red blood cells intra and post operatively. Re-look laparotomy was performed 48 h following the initial injury. This was performed by the same Trauma Surgeon with assistance from Senior Hepatobiliary and Colorectal Surgeons. Packing was removed and there was no bleeding identified. The gastric and duodenal stumps were healthy and gastro-jejunal anastomosis was performed. The transverse colonic contusion had worsened, middle colic vascular injury was identified and resection with stapled anastomosis was undertaken with defunctioning loop ileostomy. Her liver laceration was no longer bleeding and repeat systematic examination did not demonstrate further injury. After further washout her abdomen was closed.

After several days in ICU she was transferred to the ward where she remained for a period of 4 weeks. Her admission was complicated by a post-operative ileus. The humeral fracture was managed by open reduction and internal fixation. The patient was unable to recall the events of her initial trauma and multiple operations. She was an active participant in her rehabilitation and maintained good spirits throughout her admission. She received formal trauma counselling, stomal therapy education and was discharged home once deemed safe by a multidisciplinary team. She did not appear to be suffering from any long term physical or psychological disabilities as a result of her injuries. While she struggled initially with stoma management, she learned to manage this independently after several days of education. Her stoma was reversed 6 months after discharge.

Written consent was obtained from the patient prior to the creation of this report. This work has been reported in line with SCARE 2018 criteria [18].

## 3 Discussion and review of literature

Immediate transport to a tertiary trauma centre from the scene of a major incident results in increased survival outcomes [[19], [20], [21]]. A systematic, multidisciplinary approach is required for the management of major trauma. Early imaging and intervention are key factors in reducing mortality [[22], [23], [24]]. Repeated observation and evaluation of the trauma patient is vital to detect deterioration [[22], [23], [24]].

Seat belt syndrome is a recognised phenomenon associated with rapid deceleration [8,[13], [14], [15],^25^]. Seat belts are designed to re-distribute these deceleration forces to stronger points of the body. They do not decrease the forces of impact [8,[13], [14], [15],^25^]. The syndrome primarily involves soft tissue injury but laceration of liver, spleen, colon and rarely stomach have been reported in the literature [8,[13], [14], [15],^25^]. These are usually associated with use of lap seat belts rather than modern 3-point harnesses [8,[13], [14], [15],^25^]. An improperly worn seatbelt, as seen in this case, can significantly amplify the injuries described in seat belt syndrome. The injuries of our patient: left lower rib fractures, splenic laceration, gastroduodenal transection, transverse colonic injury and, liver laceration are consistent with rapid deceleration forces applied by an incorrectly worn seatbelt [26].

An extensive review of literature was conducted searching Cochrane Library, Medline and Pubmed. Duodenal injury is rare in the context of blunt trauma, and the diagnosis is delayed in up to 20 % of cases reported in some studies [[27], [28], [29], [30], [31]]. There have been several reports of duodenal injury associated with RTA and seatbelts [[32], [33], [34], [35], [36], [37], [38], [39], [40], [41], [42], [43], [44]]. Most of these occur in the second to fourth parts of the duodenum. Complete gastric transection has been described in 5 cases [[45], [46], [47], [48], [49], [50]]. All were pre-pyloric. One case presented by Carragher was associated with a vertebral Chance fracture [51]. All excepting one case required laparotomy for diagnosis and management [30]. Adequate mobilisation of the duodenum is essential to ensure injuries are not missed [29,32,52]. There were no cases of complete transection of the gastroduodenal junction in the databases identified.

Managing complex trauma often involves risk prevention and mediation. Every operative decision that is made should be done with consideration of the short- and long-term potential complications. Given the extent of intra-abdominal injury in this patient, a minimalistic approach was favoured for alimentary reconstruction. Thought was given to primary anastomosis and Roux en Y reconstruction. Gastrojejunostomy was determined to be the safest method of reconstruction of the alimentary tract with the least risk of leak.

This case adds to the body of literature and advocates for serial systematic evaluations in complex multi trauma. It demonstrates that very rare injury can be safely managed with minimalistic well known surgical reconstructive techniques once all injuries have been identified. A multi-disciplinary approach in assessment and management of trauma is vital.

## 4 Conclusion

Complex multi trauma requires swift decisive management balanced with systematic assessment to ensure optimal patient care. Seatbelts may be associated with intra-abdominal injury in rare cases but they provide an excellent means of restraint to reduce morbidity and mortality in high impact RTA when worn correctly. Traumatic gastroduodenal transection can be repaired successfully with gastrojejunostomy.

## Declaration of Competing Interest

Nil conflicts of interest.

## Funding

This case report required no funding or sponsorship.

## Ethical approval

This manuscript is a single case report and is exempt from ethics review.

## Consent

Written consent has been obtained from the patient involved in the case report.

## Author contribution

MH and PB developed the study concept together. The literature review was conducted by MH. The manuscript was originally written by MH and revised by DP, PB and CD.

## Research studies

Not applicable – single case report.

## Guarantor

Dr Matheesha Herath.

## Provenance and peer review

Not commissioned, externally peer-reviewed.
